# Markers of Cardiac Dysfunction in Cognitive Impairment and Dementia

**DOI:** 10.1097/MD.0000000000000297

**Published:** 2015-01-09

**Authors:** Saima Hilal, Yuek Ling Chai, Mohammad Kamran Ikram, Sakktivel Elangovan, Tan Boon Yeow, Xu Xin, Jun Yi Chong, Narayanaswamy Venketasubramanian, Arthur Mark Richards, Jenny P.C. Chong, Mitchell Kim Peng Lai, Christopher Chen

**Affiliations:** From the Memory, Ageing and Cognition Centre (MACC), National University Health System, Singapore (SH, YLC, MKI, SE, XX, JYC, NV, MKPL, CC); Department of Pharmacology, National University of Singapore, Singapore (SH, YLC, XX, JYC, MKPL, CC); Singapore Eye Research Institute, Singapore National Eye Center, Singapore (MKI); Department of Ophthalmology, National University of Singapore, Singapore (MKI); St. Luke's Hospital, Singapore (TBY, NV); Neuroscience Clinic, Raffles Hospital, Singapore (NV); and Cardiovascular Research Institute, National University Heart Centre, Singapore (AMR, JPCC).

## Abstract

Markers of cardiac dysfunction such as amino terminal pro-brain natriuretic peptide (NTpro-BNP) and high sensitivity cardiac troponin T (hs-cTnT) may be associated with dementia. However, limited data exist on their association with either pre-dementia stages, that is, cognitive impairment no dementia (CIND), or the burden of cerebrovascular diseases (CeVD).

We therefore, examined the association of these biomarkers of cardiac dysfunction with CeVD in both CIND and dementia.

A case–control study, with cases recruited from memory clinics and controls from memory clinics and community. All subjects underwent collection of blood samples, neuropsychological assessment, and neuroimaging. Subjects were classified as CIND and dementia based on clinical criteria whilst significant CeVD was defined as the presence of cortical infarcts and/or more than 2 lacunes and/or confluent white matter lesions in two regions of brain on Age-Related White Matter Changes Scale.

We included a total of 35 controls (mean age: 65.9 years), 78 CIND (mean age: 70.2 years) and 80 cases with dementia (mean age: 75.6 years). Plasma concentrations of hs-cTnT were associated significantly with CeVD in both CIND (odds ratios [OR]: 9.05; 95% confidence interval [CI]: 1.64–49.79) and dementia (OR: 16.89; 95%CI: 2.02–142.67). In addition, NTpro-BNP was associated with dementia with CeVD (OR: 7.74; 95%CI: 1.23–48.58). These associations were independent of other vascular risk factors.

In this study, we showed that plasma NTproBNP and hs-cTnT are associated with dementia and CIND, only when accompanied by presence of CeVD.

## INTRODUCTION

Vascular risk factors have been increasingly implicated as a cause and contributor to cognitive impairment and dementia.^1^ With respect to vascular pathology in the brain, markers of cerebral small vessel disease such as lacunar infarcts and white matter lesions are not only related to vascular dementia (VaD), but also play an important role in the development of Alzheimer's disease (AD).^2,3^ Besides systemic vascular diseases such as hypertension, the presence of cardiovascular diseases including ischemic heart disease, cardiac failure, and coronary artery disease have also been linked to impaired cognitive function.^4–7^ As these cardiac diseases are late manifestations of vascular pathology, early biomarkers such as amino terminal pro-brain natriuretic peptide (NTpro-BNP) and high sensitivity cardiac troponin T (hs-cTnT) have been investigated. These biomarkers are not only raised during overt myocardial stress and injury, but may also be increased during the early phases of vascular pathology.^8–11^

Previous studies have shown that raised levels of plasma NTpro-BNP were associated with cognitive dysfunction.^8,9,12^ However, the majority of these studies have not assessed pre-dementia stages, that is, cognitive impairment no dementia (CIND). Furthermore, data on association of these biomarkers with respect to burden of cerebrovascular diseases (CeVD) on brain magnetic resonance imaging (MRI) are lacking. Moreover, the association between other markers of cardiac dysfunction such as hs-cTnT and cognition has not been explored.

In the present study, we examined the association of plasma levels of NTpro-BNP and hs-cTnT in relation to CIND and dementia. Furthermore, we investigated their association with CeVD as determined by MRI.

## MATERIALS AND METHODS

### Study Population

For the present study, we employed a case–control design. Cases (CIND and dementia) with subjective complaints of memory loss and cognitive impairment on neuropsychological assessment were recruited from two study sites in Singapore (ie, memory clinics from National University Hospital and Saint Luke's Hospital). Controls were recruited from both memory clinics and the community (Epidemiology of Dementia in Singapore study,^13^ with a similar catchment area as cases). Controls (from memory clinic and community) were defined as those with subjective complaints of memory impairment but who were cognitively normal on objective neuropsychological assessment. All subjects underwent physical, clinical, and neuropsychological assessments and neuroimaging at the National University of Singapore (NUS) from 12 August 2010 till 25 July 2013. Ethics approval for this study was obtained from National Healthcare Group Domain-Specific Review Board (DSRB). The study was conducted in accordance with the Declaration of Helsinki. Written informed consent was obtained, in the preferred language of the participants, by bilingual study coordinators prior to their recruitment into the study.

### Examination Procedures

All the subjects underwent the following examinations.

#### Questionnaire

A detailed questionnaire was administered to collect relevant demographic and medical information. Data collected included age, gender, education, marital status, occupation, ability to live independently, handedness, previous head trauma, smoking, alcohol consumption, and family history of dementia. Previous medical history included stroke, cardiovascular diseases, hypertension, hyperlipidemia, diabetes mellitus, vitamin B 12 deficiency, thyroid disease, urinary and bowel incontinence, Parkinson's disease, depressive symptoms, and psychiatric illnesses were noted, and subsequently verified by review of the medical records. Barthel activities of daily living indices were assessed for functional status.

#### Physical Examination and Clinical Assessment

Clinical assessment included physical examination (blood pressure, pulse rate), clinical history, and the Clinical Dementia Rating Scale evaluations were performed by study clinicians.

#### Blood Tests

NTpro-BNP and hs-cTnT concentrations were measured by electrochemiluminescence immunoassay using the NTpro-BNP and hs-cTnT assay, respectively, on an automated Cobas e411 analyzer (Roche Diagnostics GmbH, Mannheim, Germany). Quality controls in both assays were below 2 standard deviations, with the range of concentration values detected in the samples from 5 to 20,627 pg/mL for NTpro-BNP and 3 to 201.50 pg/mL for hs-cTnT.

#### Neuroimaging

Magnetic resonance imaging (MRI) scans were performed on a 3T Siemens Magnetom Trio Tim scanner, using a 32-channel head coil, at the Clinical Imaging Research Centre, NUS. A number of standardized and advanced MRI brain sequences were performed to allow morphologic, microstructure and functional assessments. The sequences included T1-weighted Magnetization Prepared Rapid Gradient Recalled Echo (MPRAGE), Fluid Attenuated Inversion Recovery (FLAIR), T2-weighted and Susceptibility Weighted Imaging sequences. Scanning time was approximately 60 minutes.

MRI markers of cerebrovascular diseases were graded using the following criteria:

Cortical infarcts were defined as focal lesions with involvement of cortical gray matter, signal following CSF intensity, hyperintense rim on FLAIR images, and tissue loss of variable magnitude, with prominent adjacent sulci and ipsilateral ventricular enlargement.^14^
Lacunes were defined as lesions, 3–15 mm in diameter, with low signal on T1 weighted image and FLAIR; a high signal on T2 weighted image, and a hyperintense rim with center following the cerebrospinal fluid intensity.^14^ Lacunes were distinguished from perivascular spaces by the absence of hyperintense rim and fluid filled lesions that follow the typical course of the vessel.^13^
White matter lesions (WML) were graded using the Age Related White Matter Changes (ARWMC) scale.^15^
Significant cerebrovascular disease (CeVD) was defined as the presence of cortical infarcts and/or ≥2 lacunes and/or confluent white matter lesions in two regions of the brain (ARWMC score ≥8).

### Cognitive Impairment and Dementia Assessment

#### Neuropsychological Test Battery

Trained research psychologists administered brief cognitive tests; the Mini-Mental State Examination (MMSE) and the Montreal Cognitive Assessment (MoCA) and a formal detailed neuropsychological test battery which has been locally validated in Singapore.^16^ This battery assesses seven domains, five of which are non-memory domains.

The non-memory domains tested were:

Executive Function: Frontal Assessment Battery^17^ and Maze Task,^18^
Attention: Digit Span, Visual Memory Span^19^ and Auditory Detection,^20^
Language: Boston Naming Test^21^ and Verbal Fluency,^22^
Visuomotor speed: Symbol Digit Modality Test,^23^ Digit Cancellation,^24^
Visuoconstruction: Weschler Memory Scale—Revised (WMS-R) Visual Reproduction Copy task,^19^ Clock Drawing,^25^ and Weschler Adult Intelligence Scale—Revised (WAIS-R)subtest of Block Design.^26^

The memory domains tested are:

Verbal Memory: Word List Recall^27^ and Story Recall,
Visual Memory: Picture Recall and WMS-R Visual Reproduction.^19^

The assessment was administered in the subject's habitual language and was completed in approximately an hour.

#### Diagnosis of Cognitive Impairment and Dementia

Diagnosis of cognitive impairment and dementia were made at weekly consensus meetings attended by study clinicians and neuropsychologists. The clinical features, blood investigations, psychometrics, and neuro-images were reviewed. Cognitive impairment without dementia (CIND) was defined as impairment in at least one domain of the neuropsychological test battery using education-adjusted cutoffs of 1.5 standard deviations below established normal means on individual tests. Failure in at least half of the tests in a domain constituted failure in that domain. The etiological diagnoses of AD was made using the National Institute of Neurological and Communicative Disorders and Stroke and the Alzheimer's Disease and Related Disorders Association (NINCDS-ADRDA) and the National Institute of Neurological Disorders and Stroke and Association Internationale pour la Recherché et l’ Enseignement en Neurosciences (NINDS-AIREN) criteria for VaD and mixed dementia.^28,29^

#### Covariates

Risk factors such as hypertension and cardiovascular diseases were collected from physical and clinical interview and medical records and classified as present or absent. Hypertension was defined as systolic blood pressure ≥140 mm Hg and/or diastolic blood pressure ≥90 mm Hg or use of antihypertensive medications. Cardiovascular diseases were classified as a previous history of atrial fibrillation, congestive heart failure and myocardial infarction. Education was categorized as low education (years of education ≤6) and higher education (years of education >6).

### Statistical Analysis

Statistical analysis was performed using standard statistical software (Statistical Package for Social Science, SPSS V21, SPSS, Inc., Armonk, New York, USA). Analyses of covariance or Chi-square test were used to compare the characteristics of the cases and controls groups. The levels of biomarkers of cardiac dysfunction were included as a determinant in the logistic models and were categorized into tertiles in order to secure enough numbers in each category. Dementia and its preclinical stages were taken as outcome. Multiple logistic regression analysis with odds ratios (OR) and 95% confidence interval (CI) were computed initially for CIND and dementia. Further regression analysis was then constructed for both CIND and dementia stratified by CeVD on MRI. The models were initially adjusted for age, gender, education, and additionally for hypertension and cardiovascular diseases. *P* value <0.05 was considered significant.

## RESULTS

A total of 259 subjects (49 controls, 98 CIND, and 112 dementia) were recruited into this study, of whom sufficient blood samples were available in 193. Table 1 shows the baseline characteristics of the controls (n = 35) and subjects with CIND (n = 78) or dementia (n = 80). Compared to controls, subjects with CIND or dementia were older, had lower education, and had higher prevalence of hypertension and diabetes. Among the 78 subjects with CIND, 37 were classified as having significant CeVD on MRI. For the 80 cases with dementia, there were 46 subjects with significant CeVD, that is, 31 AD and 15 VaD. Plasma levels of these markers of cardiac dysfunction were raised in individuals with atrial fibrillation, myocardial infraction and congestive heart failure.

**TABLE 1:**
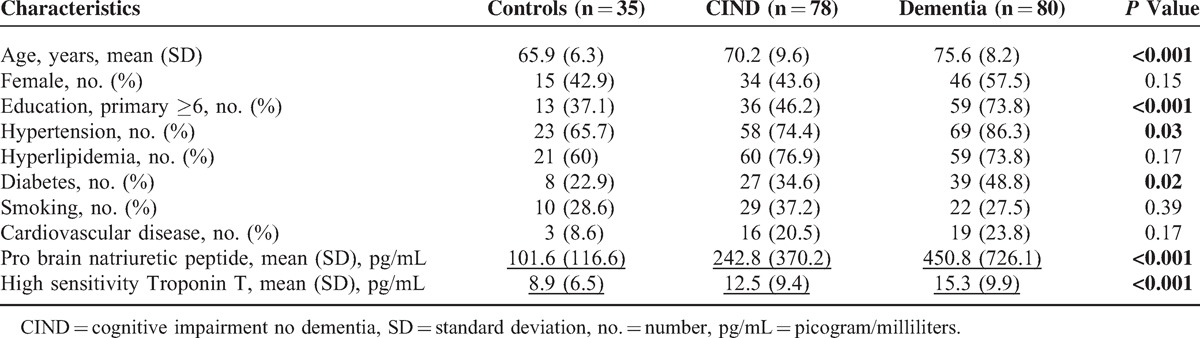
Baseline Characteristics of the Participants Based on Their Cognitive Categories (n = 193)

Table 2 shows the association between markers of cardiac dysfunction and cognitive impairment. A trend of association was observed from CIND to dementia with higher tertiles of NTpro-BNP and hs-cTnT after adjustment for age, gender, education, hypertension, and cardiovascular diseases. However, these associations remained non-significant for both the markers of cardiac dysfunction with cognition.

**TABLE 2:**
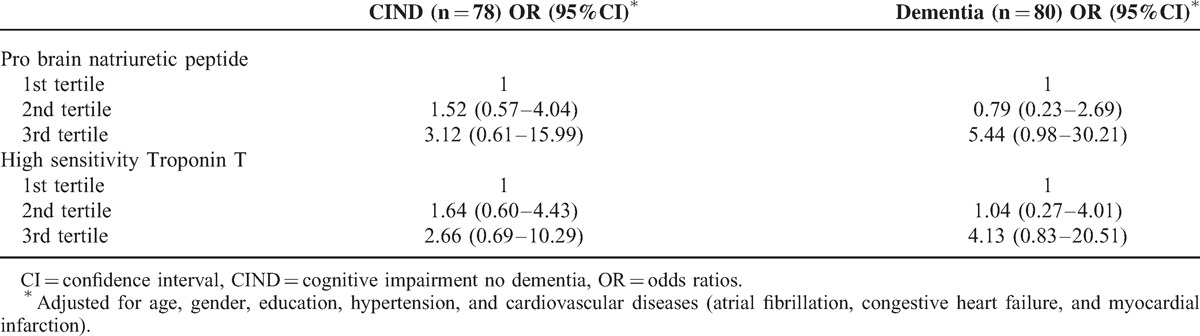
The Association Between Markers of Cardiac Dysfunction (Tertiles) and Cognitive Impairment Expressed as Odds Ratios With 95% Confidence Intervals

Table 3 shows the relationship of markers of cardiac dysfunction with CIND and dementia stratified by presence and absence of CeVD. Higher tertiles of hs-cTnT was associated with both cognitive impairment and dementia in patients with significant CeVD whereas the higher tertiles of NTpro-BNP was only associated with dementia with significant CeVD. These associations remain unaltered after correcting for all the covariates. Further adjustment with age^2^ yielded similar association.

**TABLE 3:**
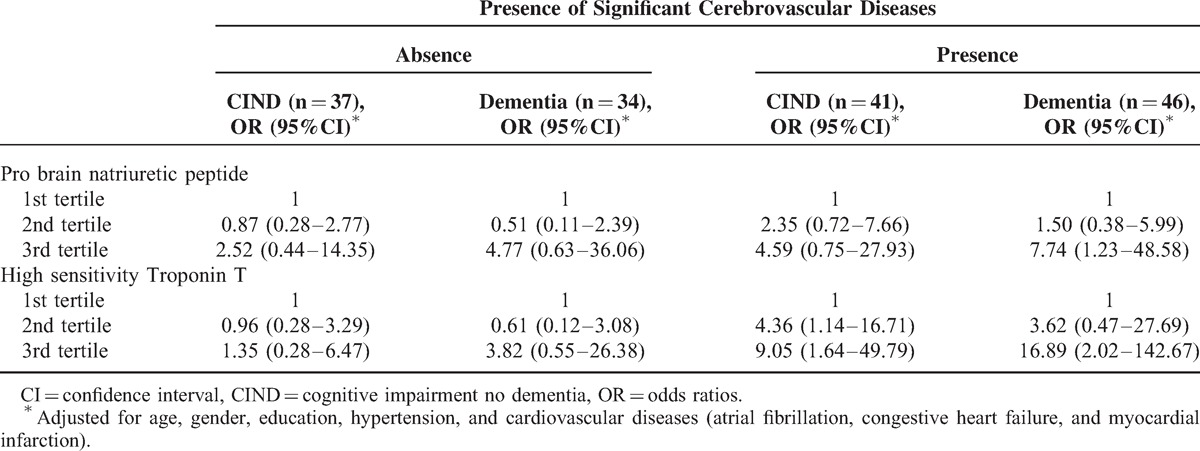
The Association Between Markers of Cardiac Dysfunction (Tertiles) and Cognitive Impairment Stratified by Significant Cerebrovascular Diseases Expressed as Odds Ratios With 95% Confidence Intervals

## DISCUSSION

In this study, we found that the biomarkers of cardiac disease such as NTpro-BNP and hs-cTnT were associated with cognitive impairment and dementia in patients with significant CeVD, independent of other vascular risk factors.

Coronary heart disease and congestive heart failure have been previously associated with impaired cognitive function.^30–32^ However, by the time cognitive impairment becomes clinically apparent significant cerebral damage may have already occurred in the brain.^33,34^ Hence, early markers of cardiac dysfunction such as NT-proBNP and hs-cTnT are of interest so that timely interventions may be employed. NT-proBNP is secreted from the cardiomyocytes primarily in response to cardiomyocyte stretch whereas hs-cTnT is a sensitive and specific marker for myocardial injury with established clinical application in the diagnosis of acute myocardial infarction. Several studies have shown that NT-proBNP is associated with cognitive decline and dementia.^9,35,36^ These associations remain unaltered in individuals with^8^ and without history of cardiovascular risk factors.^35^ With respect to pre-dementia stages, there is some data to suggest that NT-proBNP was associated with progression from mild cognitive impairment to AD.^37^ Moreover, for the subtypes of dementia, two studies showed a stronger association with VaD compared to AD.^9,12^ Nevertheless, these studies were limited by either none or low resolution (1.5T) MRI neuroimaging to objectively supplement the diagnosis of dementia and a neglect of the pre-dementia stages of cognitive impairment. Furthermore, there are no previous reports on the association of hs-cTnT with cognitive impairment and dementia.

Our study adds further to the existing literature by showing significant association of markers of cardiac dysfunction with cognitive impairment only in the presence of CeVD. It has been suggested that the left ventricular dysfunction and ischemic heart disease not only releases these biomarkers but also activates several other inflammatory markers which leads to ischemic damage in regions selective to cognitive function.^38^ NT-proBNP has been previously correlated with silent brain infarcts and white matter lesions in individuals with subclinical brain injury.^10^ Moreover, higher level of NT-proBNP is also linked to atrial fibrillation^39^ which in turn is associated with cardioembolic stroke^40^ and reduced cognitive function.^41^ Our findings thus are consistent with those reported previously where NT-proBNP was significantly associated with VaD^9,12^ and AD with CeVD indicating significant cerebrovascular damage to the brain. These associations remain significant after accounting for atrial fibrillation, ischemic heart disease, and congestive heart failure.

Our study has several limitations. First, as these data were examined cross-sectionally it is not possible to establish the temporal association between these markers of cardiac dysfunction and the development of cognitive impairment. Second, we were unable to examine the association with VaD separately due to small numbers. Third, cases and half of the controls were derived from 2 locations, memory clinic and community, although representative of the elderly population in Singapore. The control group was relatively younger and had less burden of CeVD compared to cognitively impaired individuals which could have resulted in selection bias and residual confounding. Also there is a higher burden of vascular risk factors (hypertension, hyperlipidemia, and diabetes) in our sample which limits generalizability of the results to the general population. Fourth, due to lack of power it is possible that we were unable to detect the associations of these markers of cardiac dysfunction with CIND and dementia without the presence of CeVD. Strengths of the study include; extensive neuropsychological assessment to diagnose cognitive impairment and dementia, availability of 3T neuroimaging to grade and classify individuals with significant CeVD.

## CONCLUSIONS

In conclusion, we showed that subjects with higher concentrations of NTpro-BNP and hs-cTnT were more likely to be cognitively impaired only in the presence of cerebrovascular disease independent of vascular risk factors. This suggests that raised markers of cardiac dysfunction may help identify patients at risk for developing cerebrovascular disease-associated cognitive decline and dementia. Our study provides further insights into the pathophysiology of cognitive impairment and dementia for which vascular pathology has long been implicated. Further prospective studies are needed to unravel the additional value of markers of cardiac dysfunction in predicting cognitive decline and if they may serve as surrogate outcomes in evaluating the effectiveness of novel cerebrovascular disease related dementia treatments.

## References

[1] Kling MA Trojanowski JQ Wolk DA Vascular disease and dementias: paradigm shifts to drive research in new directions. *Alzheimers Dement* 2013; 9:76–92. 23183137 10.1016/j.jalz.2012.02.007 PMC3640817

[2] Prins ND van Dijk EJ den Heijer T Cerebral small-vessel disease and decline in information processing speed, executive function and memory. *Brain* 2005; 128:2034–2041. 15947059 10.1093/brain/awh553

[3] Pantoni L Poggesi A Inzitari D Cognitive decline and dementia related to cerebrovascular diseases: some evidence and concepts. *Cerebrovasc Dis* 2009; 27:191–196. 19342851 10.1159/000200459

[4] Pressler SJ Cognitive functioning and chronic heart failure: a review of the literature (2002–July 2007). *J Cardiovasc Nurs* 2008; 23:239–249. 18437066 10.1097/01.JCN.0000305096.09710.ec

[5] Muqtadar H Testai FD Gorelick PB The dementia of cardiac disease. *Curr Cardiol Rep* 2012; 14:732–740. 22968344 10.1007/s11886-012-0304-8

[6] Dziedzic T Systemic inflammatory markers and risk of dementia. *Am J Alzheimer's Dis Dement* 2006; 21:258–262. 10.1177/1533317506289260 PMC10833275 16948290

[7] van den Hurk K Reijmer YD van den Berg E Heart failure and cognitive function in the general population: the Hoorn Study. *Eur J Heart Fail* 2011; 13:1362–1369. 21990341 10.1093/eurjhf/hfr138

[8] Gunstad J Poppas A Smeal S Relation of brain natriuretic peptide levels to cognitive dysfunction in adults >55 years of age with cardiovascular disease. *Am J Cardiol* 2006; 98:538–540. 16893713 10.1016/j.amjcard.2006.02.062 PMC2748274

[9] Kerola T Nieminen T Hartikainen S B-type natriuretic peptide as a predictor of declining cognitive function and dementia—a cohort study of an elderly general population with a 5-year follow-up. *Ann Med* 2010; 42:207–215. 20384435 10.3109/07853891003652542

[10] Dadu RT Fornage M Virani SS Cardiovascular biomarkers and subclinical brain disease in the atherosclerosis risk in communities study. *Stroke* 2013; 44:1803–1808. 23660848 10.1161/STROKEAHA.113.001128 PMC4334904

[11] Webb IG Yam ST Cooke R Elevated baseline cardiac troponin levels in the elderly—another variable to consider? *Heart Lung Circ* 2014; doi: 10.1016/j.hlc.2014.07.071. 10.1016/j.hlc.2014.07.071 25168154

[12] Kondziella D Gothlin M Fu M B-type natriuretic peptide plasma levels are elevated in subcortical vascular dementia. *Neuroreport* 2009; 20:825–827. 19424098 10.1097/WNR.0b013e328326f82f

[13] Hilal S Ikram MK Saini M Prevalence of cognitive impairment in Chinese: epidemiology of dementia in Singapore study. *J Neurol Neurosurg Psychiatry* 2013; 84:686–692. 23385846 10.1136/jnnp-2012-304080

[14] Wardlaw JM Smith EE Biessels GJ Neuroimaging standards for research into small vessel disease and its contribution to ageing and neurodegeneration. *Lancet Neurol* 2013; 12:822–838. 23867200 10.1016/S1474-4422(13)70124-8 PMC3714437

[15] Kapeller P Barber R Vermeulen RJ Visual rating of age-related white matter changes on magnetic resonance imaging: scale comparison, interrater agreement, and correlations with quantitative measurements. *Stroke* 2003; 34:441–445. 12574557 10.1161/01.str.0000049766.26453.e9

[16] Yeo D Gabriel C Chen C Pilot validation of a customized neuropsychological battery in elderly Singaporeans. *Neurol J South East Asia* 1997 123.

[17] Dubois B Slachevsky A Litvan I Pillon B The FAB: a frontal assessment battery at bedside. *Neurology* 2000; 55:1621–1626. 11113214 10.1212/wnl.55.11.1621

[18] Porteus SD The Maze Test and Clinical Psychology. Palo Alto, CA: Pacific Books; 1959.

[19] Wechsler D Wechsler Memory Scale—Revised. 3rd ed San Antonio TX: Jovanovich; 1997.

[20] Lewis RF Rennick PM Manual for the Repeatable Cognitive Perceptual-Motor Battery. Clinton Township, MI: Axon; 1979.

[21] Mack WJ Freed DM Williams BW Henderson VW Boston Naming Test: shortened versions for use in Alzheimer's disease. *J Gerontol* 1992; 47:154–158. 10.1093/geronj/47.3.p154 1573197

[22] Isaacs B Kennie AT The Set test as an aid to the detection of dementia in old people. *Br J Psychiatry* 1973; 123:467–470. 4748864 10.1192/bjp.123.4.467

[23] Smith A Symbol Digit Modalities Test. Los Angeles, CA: Western psychological service; 1973.

[24] Diller L Ben-Yishay Y Gerstman LJ Studies in Cognition and Rehabilitation in Hemiplegia. New York: New York University Medical Center Institute of Rehabilitation Medicine; 1974.

[25] Sunderland T Hill JL Mellow AM Clock drawing in Alzheimer's disease. A novel measure of dementia severity. *J Am Geriatr Soc* 1989; 37:725–729. 2754157 10.1111/j.1532-5415.1989.tb02233.x

[26] Wechsler D Wechsler Adult Intelligence Scale—Revised. San Antonio, TX: Harcourt Brace Jovanovich; 1981.

[27] Sahadevan S Tan NJ Tan T Tan S Cognitive testing of elderly Chinese people in Singapore: influence of education and age on normative scores. *Age Ageing* 1997; 26:481–486. 9466300 10.1093/ageing/26.6.481

[28] Roman GC Tatemichi TK Erkinjuntti T Vascular dementia: diagnostic criteria for research studies. Report of the NINDS-AIREN International Workshop. *Neurology* 1993; 43:250–260. 8094895 10.1212/wnl.43.2.250

[29] McKhann GM Knopman DS Chertkow H The diagnosis of dementia due to Alzheimer's disease: recommendations from the National Institute on Aging-Alzheimer's Association workgroups on diagnostic guidelines for Alzheimer's disease. *Alzheimer's Dement* 2011; 7:263–269. 21514250 10.1016/j.jalz.2011.03.005 PMC3312024

[30] Cohen RA Moser DJ Clark MM Neurocognitive functioning and improvement in quality of life following participation in cardiac rehabilitation. *Am J Cardiol* 1999; 83:1374–1378. 10235098 10.1016/s0002-9149(99)00103-4

[31] Vogels RL Oosterman JM van Harten B Profile of cognitive impairment in chronic heart failure. *J Am Geriatr Soc* 2007; 55:1764–1770. 17727641 10.1111/j.1532-5415.2007.01395.x

[32] Hoth KF Poppas A Moser DJ Cardiac dysfunction and cognition in older adults with heart failure. *Cogn Behav Neurol* 2008; 21:65–72. 18541980 10.1097/WNN.0b013e3181799dc8

[33] Bell-McGinty S Lopez OL Meltzer CC Differential cortical atrophy in subgroups of mild cognitive impairment. *Arch Neurol* 2005; 62:1393–1397. 16157746 10.1001/archneur.62.9.1393

[34] Saykin AJ Wishart HA Rabin LA Older adults with cognitive complaints show brain atrophy similar to that of amnestic MCI. *Neurology* 2006; 67:834–842. 16966547 10.1212/01.wnl.0000234032.77541.a2 PMC3488276

[35] Naito J Naka Y Watanabe H Clinical impression of brain natriuretic peptide levels in demented patients without cardiovascular disease. *Geriatr Gerontol Int* 2009; 9:242–245. 19702933 10.1111/j.1447-0594.2009.00526.x

[36] Daniels LB Laughlin GA Kritz-Silverstein D Elevated natriuretic peptide levels and cognitive function in community-dwelling older adults. *Am J Med* 2011; 124:670 e671–e678. 21683832 10.1016/j.amjmed.2011.02.027 PMC3173742

[37] Buerger K Uspenskaya O Hartmann O Prediction of Alzheimer's disease using midregional proadrenomedullin and midregional proatrial natriuretic peptide: a retrospective analysis of 134 patients with mild cognitive impairment. *J Clin Psychiatry* 2011; 72:556–563. 21208578 10.4088/JCP.09m05872oli

[38] Angermann CE Frey A Ertl G Cognition matters in cardiovascular disease and heart failure. *Eur Heart J* 2012; 33:1721–1723. 22645187 10.1093/eurheartj/ehs128

[39] Richards M Di Somma S Mueller C Atrial fibrillation impairs the diagnostic performance of cardiac natriuretic peptides in dyspneic patients: results from the BACH Study (Biomarkers in ACute Heart Failure) JACC. *Heart Fail* 2013; 1:192–199. 10.1016/j.jchf.2013.02.004 24621869

[40] Rost NS Biffi A Cloonan L Brain natriuretic peptide predicts functional outcome in ischemic stroke. *Stroke* 2012; 43:441–445. 22116811 10.1161/STROKEAHA.111.629212 PMC3265658

[41] Chong AY Blann AD Patel J Endothelial dysfunction and damage in congestive heart failure: relation of flow-mediated dilation to circulating endothelial cells, plasma indexes of endothelial damage, and brain natriuretic peptide. *Circulation* 2004; 110:1794–1798. 15364797 10.1161/01.CIR.0000143073.60937.50

